# Ecological Importance of Small-Diameter Trees to the Structure, Diversity and Biomass of a Tropical Evergreen Forest at Rabi, Gabon

**DOI:** 10.1371/journal.pone.0154988

**Published:** 2016-05-17

**Authors:** Hervé R. Memiaghe, James A. Lutz, Lisa Korte, Alfonso Alonso, David Kenfack

**Affiliations:** 1 Institut de Recherche en Écologie Tropicale (IRET), Libreville, Gabon; 2 Wildland Resources Department, Utah State University, Logan, Utah, United States of America; 3 Center for Conservation and Sustainability, Smithsonian Conservation Biology Institute, Gamba, Gabon; 4 Center for Conservation and Sustainability, Smithsonian Conservation Biology Institute, Washington DC, United States of America; 5 Center for Tropical Forest Science -Forest Global Earth Observatory, Smithsonian Tropical Research Institute, Smithsonian Institution, Washington DC, United States of America; Chinese Academy of Forestry, CHINA

## Abstract

Tropical forests have long been recognized for their biodiversity and ecosystem services. Despite their importance, tropical forests, and particularly those of central Africa, remain understudied. Until recently, most forest inventories in Central Africa have focused on trees ≥10 cm in diameter, even though several studies have shown that small-diameter tree population may be important to demographic rates and nutrient cycling. To determine the ecological importance of small-diameter trees in central African forests, we used data from a 25-ha permanent plot that we established in the rainforest of Gabon to study the diversity and dynamics of these forests. Within the plot, we censused 175,830 trees ≥1 cm dbh from 54 families, 192 genera, and 345 species. Average tree density was 7,026 trees/ha, basal area 31.64 m^2^/ha, and above-ground biomass 369.40 Mg/ha. Fabaceae, Ebenaceae and Euphorbiaceae were the most important families by basal area, density and above-ground biomass. Small-diameter trees (1 cm ≥ dbh <10 cm) comprised 93.7% of the total tree population, 16.5% of basal area, and 4.8% of the above-ground biomass. They also had diversity 18% higher at family level, 34% higher at genus level, and 42% higher at species level than trees ≥10 cm dbh. Although the relative contribution of small-diameter trees to biomass was comparable to other forests globally, their contribution to forest density, and diversity was disproportionately higher. The high levels of diversity within small-diameter classes may give these forests high levels of structural resilience to anthropogenic/natural disturbance and a changing climate.

## Introduction

**Fig 1:**
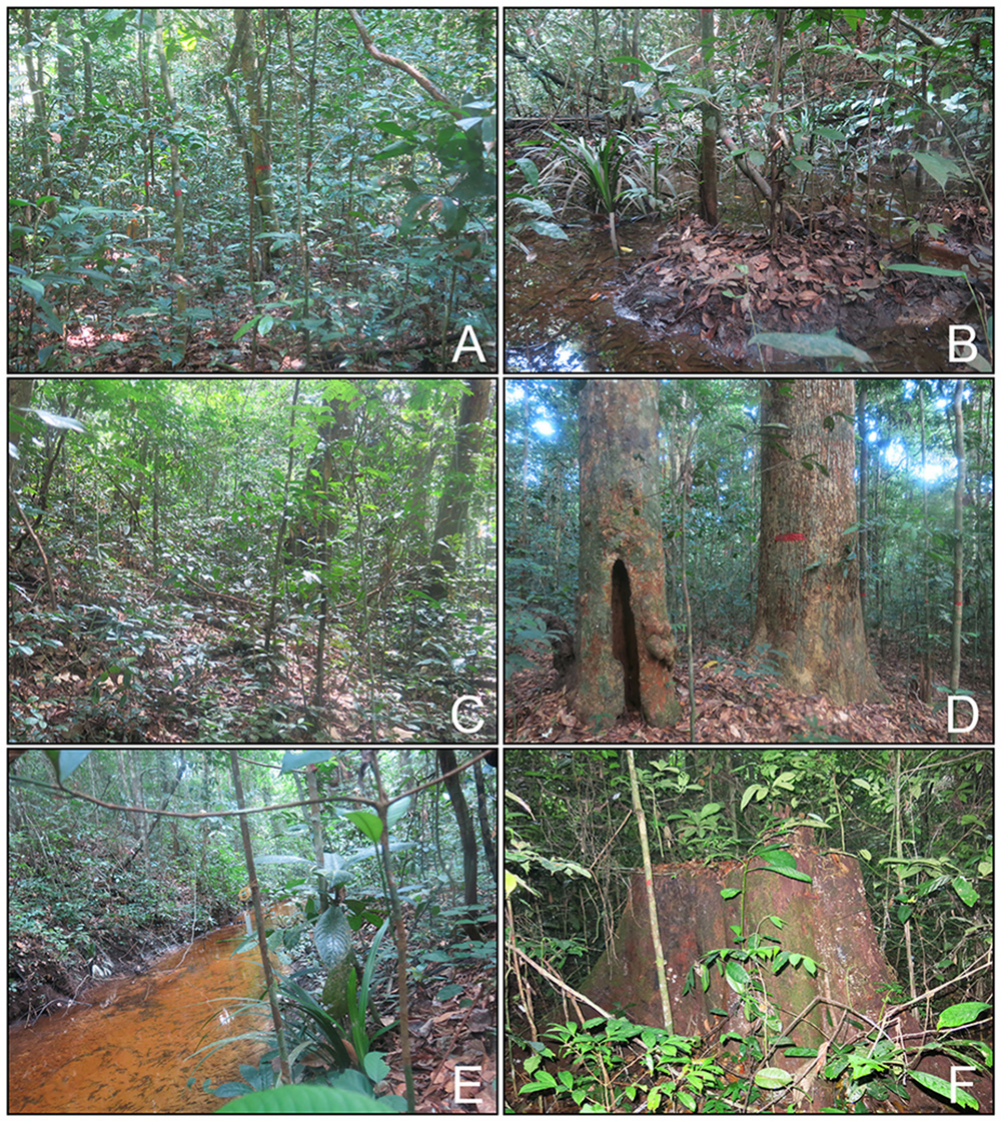
Representative photos of the four strata of the forest canopy. (A) Treelets (<10 m tall). (B) Understory trees (10 m to 20 m tall), (C) lower canopy trees (20 m to 30 m tall) and (D) upper canopy trees (≥30 m tall). (E) The plot also features a stream and 11 stumps from past logging of valuable trees (F).

Tropical forests have long been recognized for their biodiversity and ecosystem services such as carbon sequestration, habitat provision for vertebrates and invertebrates, and non-timber resources [1–3]. The tropical forest biome extends across three floristic regions—Central and South America, the southeastern Asian-Pacific, and equatorial Africa, all with high biodiversity. The African tropical rainforest has been least studied, and information is lacking regarding tree species diversity and the distribution of structural elements responsible for carbon storage and other ecosystem functions. Because small-diameter trees (here defined as those with less than 10 cm diameter at breast height [dbh, 1.3 m above the ground] and greater than or equal to 1 cm in diameter) are such a visually prevalent element of these forests (Fig 1), we predict that as in other African forests, they would be a much more important component of the forest diversity, structure and biomass than other forests globally.

The small-diameter tree population has often been overlooked because it is not important to timber extraction, and is a much smaller constituent of forest biomass than the larger trees (e.g., [4]). Until recently, most studies in Africa concentrated on large-diameter trees (here defined as those with dbh ≥10 cm; e.g., [5–8]. However, recent studies have shown that the small-diameter tree population may be important to demographic rates and nutrient cycling (e.g., [9]), and that the relative contribution of smaller diameter trees to forest biomass may be higher in tropical Africa than in other tropical and temperate forests (e.g., [10]). Small-diameter trees may also contribute disproportionately to woody plant diversity [11], as many taxa that reach 1 cm dbh are not present at the 10 cm diameter class—potentially much more so in tropical Africa than in other forests.

We, therefore, sought to establish a permanent research plot where we could study the unique contributions of smaller diameter trees to the distribution and abundance of all trees, and also to compare the Rabi forest with others in the Smithsonian Center for Tropical Forest Science ForestGEO network of long-term permanent plots [12], including those also located in the Congo basin rainforest—Ituri in Democratic Republic Congo [13], Korup in Cameroon [14], and at Rabi in Gabon (the present study). Previous results from Ituri and Korup showed that tropical African forests have some of the highest proportions of small-diameter trees in the world, both in terms of forest density and contributions to overall biomass [13,15,16]. Similarly, Lin et al. [17] showed that small-diameter trees (1 cm ≤ dbh <10 cm) contributed 10.4% of the biomass in a subtropical forest in China, and Vincent et al. [18] showed that the presence of small-diameter trees contributes to higher local levels of aboveground biomass. Our objectives were to characterize the composition and structure of the forests of Rabi and examine the relative contribution of small-diameter trees to tree abundance, basal area, above-ground biomass, and diversity. We hypothesized that the relative importance of small-diameter trees would be higher than in other forests globally and, therefore, that a comprehensive understanding of tropical African forests requires sampling of small-diameter trees in addition to the current emphasis on larger diameter individuals.

## Methods

The research authorization to carry out this study was granted by the Government of Gabon, through the Centre National de la Recherche Scientifique et Technologique (CENAREST).

### Study site

**Fig 2:**
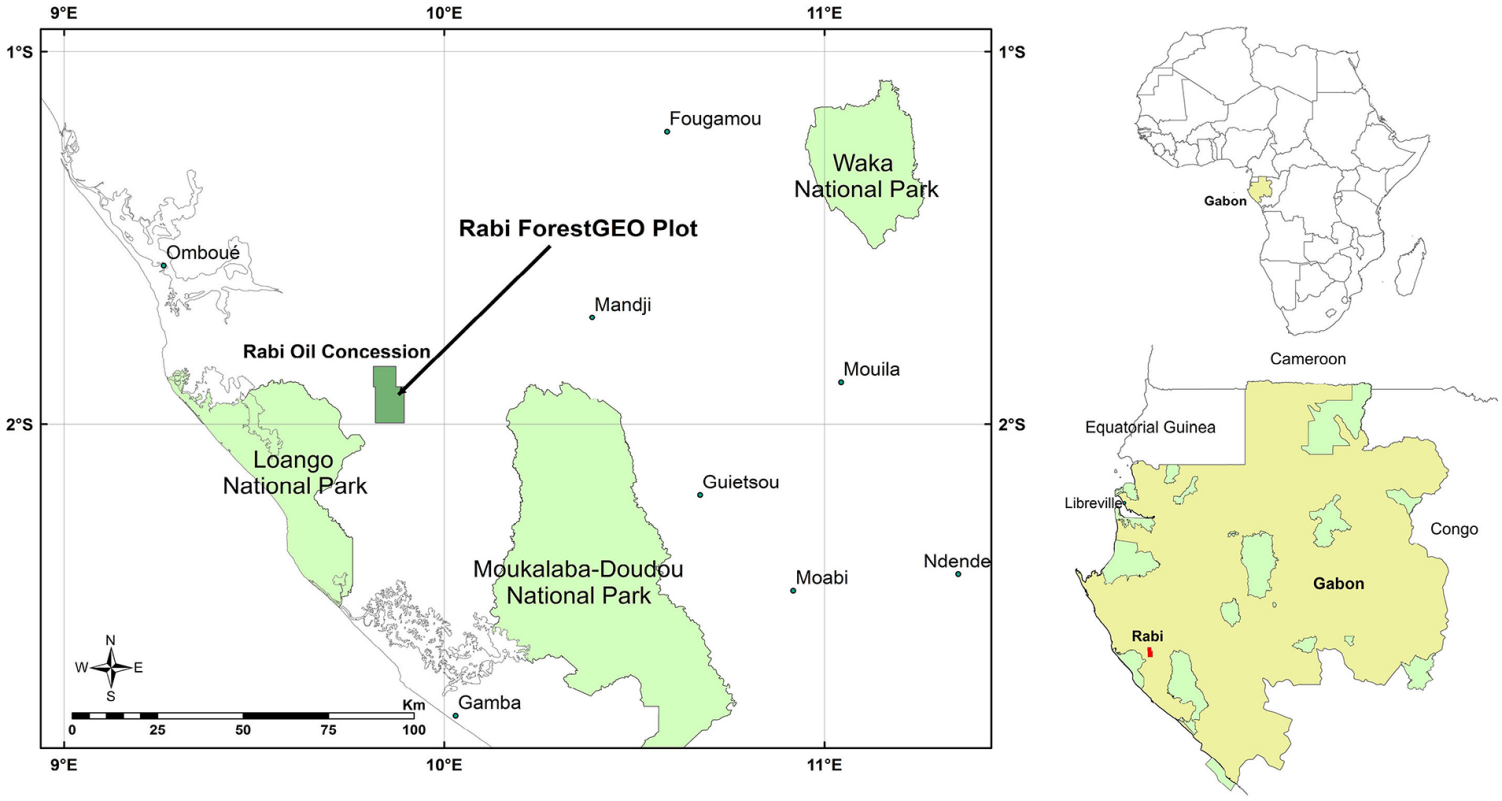
Map of the Rabi Forest Monitoring Plot in relation to the Gamba Complex of Protected Areas and the Rabi Oil Concession (Africa and Gabon inset, right).

The study site is in the Gamba Complex of Protected Areas (1°50′ to 3°10′ S; 9° 15′ to10° 50′ E) in southwestern Gabon (Fig 2). This region is the southern portion of Guineo-Congolian forest type [14,19–21], which includes swamp and mixed moist semi-evergreen forest types [22]. The Gamba Complex includes two national parks, Loango National Park on the west and Moukalaba Doudou National Park on the east. This area is the largest protected area in Gabon, covering 267,667 km^2^ or about 4% of the total area of Gabon [20].

Rabi is in the northern part of the Gamba Complex between the two national parks. The vegetation is characterized by the abundance of the tree species such as *Dichostemma glaucescens*, *Diogoa zenkeri*, *Klaineanthus gaboniae*, *Coula edulis*, *Crudia gabonensis* and *Odyendea gabonensis*. The most abundant families are Fabaceae, Euphorbiaceae and Olacaceae [21,23]. Family classification follows the Angiosperm Phylogeny Group III [24].

Soils are characterized as ferralitic and hydromorphic, with mostly sandy clay (approximately 25% clay) to clay sand (approximately 35% clay) [20]. Temperature throughout the year is almost invariant between 24°C and 28°C [21]. Annual precipitation averages 2299 mm. There are two main seasons, a dry season from June to September with precipitations averaging 24 mm/month and a wet season between October and May when precipitations average 269 mm/month. November and March with averages of 406 mm and 303 mm of rain respectively are the wettest months of the year, while July with an average of 11 mm is the driest month of the year (Shell Gabon unpublished data, 1985 to 2015).

**Fig 3:**
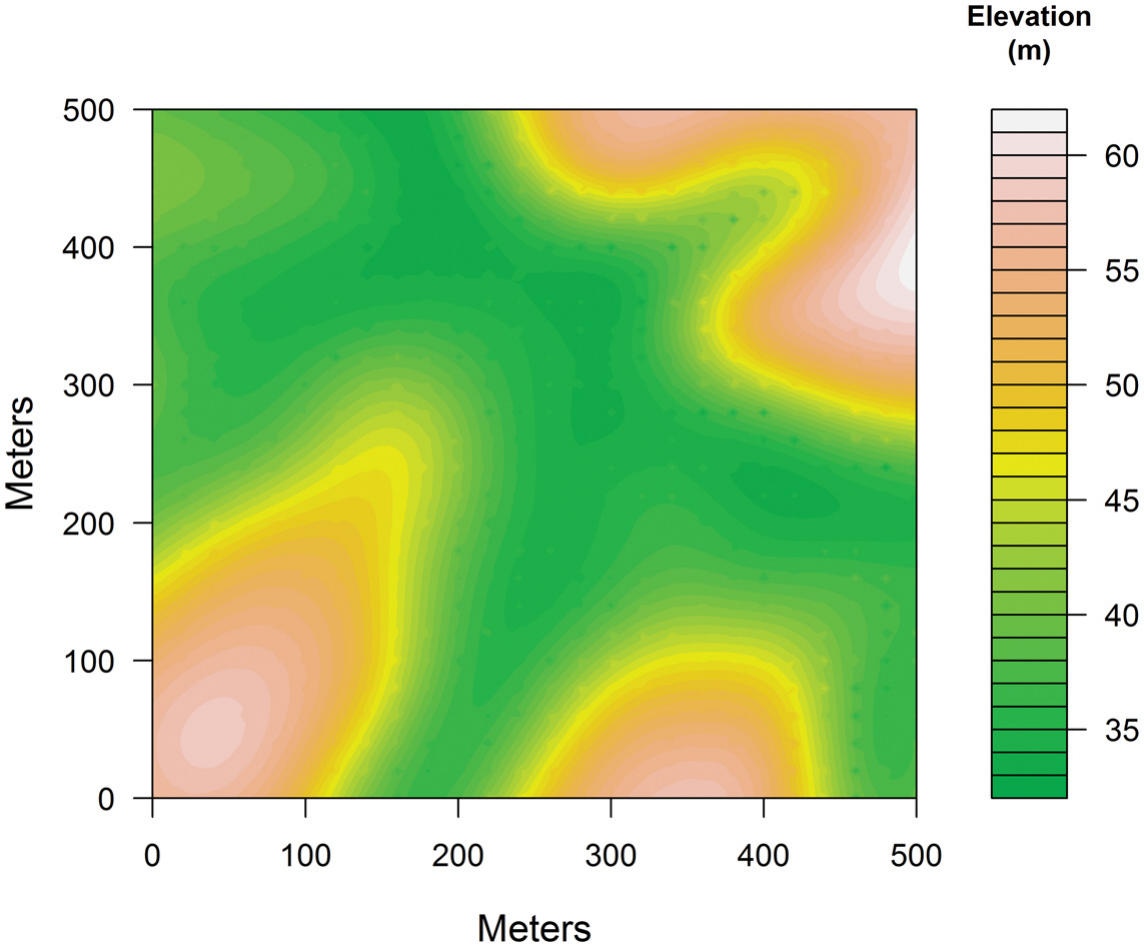
Topographic map of the 25-ha Rabi plot with 1-m contour intervals. The river flows at the lowest elevations, specifically between the 32-m and 36-m contours.

The 25-ha permanent plot (plot center 1°55′S, 9°52′W) is orientated southeast—northwest (Fig 3). Plot elevation varies between 32 m and 62 m, and the plot is bisected by a stream surrounded by gentle slopes and three ridges (Fig 3). The forest in the area underwent selective logging, mostly of *Lophira alata* (Azobe), prior to 1990; there are 11 stumps from felled trees inside the plot (Fig 1F). In addition to the selective logging, seismic assessments were conducted in the area in the 1980s. Seismic assessments involve the temporary use of heavy equipment in localized areas resulting in removal of smaller statured vegetation [25,26]. The forest canopy is generally divided into four strata—treelets, understory, lower canopy trees and upper canopy (Fig 1).

### Data collection

Field methods followed the standards of the Smithsonian Center for Tropical Forest Science [27]. The 25-ha was divided into 625 quadrats of 20 m × 20 m each. For tree enumeration, each quadrat was in turn divided into 16 subquadrats of 5 m × 5 m. Within each subquadrat, the main stem of every tree ≥1 cm in diameter at breast height (dbh; 1.3 m above ground level) was mapped (subsidiary stems of the same individual were not mapped). If the tree had either an irregularity or a buttress at 1.3 m height, the height of measurement was moved (and recorded) to avoid the irregularity or the buttress.

Tree enumeration (diameter measurement, tagging and mapping) was carried out from June 2010 to June 2012. Tree identification was conducted in the field and in herbaria. In the field, trees were grouped into morphospecies—morphologically identical entities—mostly on the basis of vegetative characters. To ensure consistency in the groupings, up to 15 voucher specimens were collected for each morphospecies and compared side-by-side. Over time (2012–2014), flowering and fruiting material were collected for 85% of the morphospecies for further taxonomic identifications at herbaria in Libreville, Leiden and Brussels. Voucher specimens for the Rabi plot are deposited at the National Herbarium of Gabon, the Naturalis Biodiversity Center in Leiden, the Missouri Botanical Garden and the Smithsonian Museum of Natural History.

### Maximum height at maturity

The classification of Rabi species into life-forms followed Kenfack et al. [14]. Each species was assigned to one of the following four life-forms according to the maximum height that they attain at maturity, using information from the literature and our field observations. Trees that do not normally reach 10 m in height and are <10 cm dbh were classified as treelets; understory trees were those 10 m to 20 m tall and 10 cm to 30 cm dbh; lower canopy included trees 20 m to 30 m tall and 30 cm to 60 cm dbh; and finally, upper canopy trees were defined as those reaching >30 m in height and >60 cm dbh.

### Data Analyses

Because most forest inventory plots in Central Africa are 1 ha in area and include only trees ≥10 cm dbh, for comparison we calculated abundances, basal area, Fisher’s diversity and aboveground biomass (AGB) per hectare and for two diameter classes; 1 cm ≤ dbh <10 cm and ≥10 cm. Diversity was analyzed at the levels of families, genera and species. Aboveground biomass was estimated for each individual tree (including all stems for multi-stemmed trees) using the dbh and the wood density allometric equation

$$\text{AGB}=\rho \times \text{exp}\left(-1.499+2.148\times lnD+0.207\times lnD^{2}-0.0281\times lnD^{3}\right)$$

from Chave et al. [28], where AGB is aboveground dry biomass (in kg), ρ the wood density (g/cm3), D the dbh (in cm), ln the natural logarithm, and exp the exponential function. Frequency was calculated based on the 20 m × 20m quadrats that make up the plot. Abundance-diameter relationships were calculated with 1 cm bins. All analyses were performed using version 3.4.2 of R [29] and the CTFS R package (http://ctfs.arnarb.harvard.edu/Public/CTFSRPackage/index.php/web).

## Results

### Floristics and diversity

We found 345 morphospecies within the 25-ha plot, of which 294 (85.2%) were identified to species and the remaining 51 (14.8%) to genus level. Six of the species identified to genus level have been confirmed to be new to science, and we expect that others will be confirmed as new to science when fertile material can be collected for these morphospecies. Three species in the plot were recorded in Gabon for the first time, *Okoubaka aubrevillei* Pellegr (Santalaceae) & Normand, *Magnistipula multinervia* Burgt (Chrysobalanaceae), and *Beilschmiedia auriculata* Robyns & R. Wilczek (Lauraceae). Because field identification was carried out for some portions of the plot several months after tree enumeration and mapping, a total of 2,109 individual trees were dead when they were revisited and not identified nor included in the analysis.

The 345 morphospecies belong to 54 families and 192 genera. Rubiaceae, with 57 species in 32 genera, was the most diverse family, followed by Fabaceae that had 43 species in 26 genera. Other diverse families in the plot included Annonaceae (17 species in 9 genera), Phyllanthaceae (16 species in 10 genera) and Anacardiaceae (16 species in 2 genera) (see S1 Appendix for a complete listing of species). The genera *Trichoscypha* and *Diospyros* were the richest with 14 and 12 species respectively, followed by *Beilschmiedia* (8), *Maesobotrya*, *Memecylon*, and *Xylopia*, each with seven species. Of these genera, *Trichoscypha*, *Beilschmiedia* and *Memecylon* had the highest number of unidentified species (S1 Appendix).

The small-diameter tree group comprised 333 species (96% of the total) in 185 genera and 52 families, while 233 species in 137 genera and 44 families were recorded among large-diameter trees. There were 207 species/ha for all trees in the plot, 201 species/ha for small-diameter trees, as compared to only 84 species/ha for large-diameter trees (Table 1). Fisher’s α for all trees was 40.1 per ha, almost the same (39.4/ha) for small-diameter trees, but lower for large diameter-trees (30.8/ha).

**Table 1:** Comparison of the small-diameter (1 cm ≥ dbh <10 cm) and large-diameter (dbh ≥10cm) tree contribution to structure and diversity of the Rabi 25-ha plot. Numbers in parenthesis represent standard deviation or percentages.

	≥ 1 cm	<10 cm	≥ 10 cm
All families	54	52 (96.3%)	44 (81.5%)
All genera	194	187 (96.4%)	139 (71.6%)
All species	345	333 (96.5%)	234 (67.8%)
All trees	175,660	164,491 (93.6%)	11,170 (6.4%)
Total Basal area (m^2^)	791.23	130.73 (16.5%)	660.60 (83.5%)
Total aboveground biomass (Mg)	9,235.18	458.32 (5.0%)	8,777.01 (95.0%)
Mean density per ha	7026 (660)	6,580 (644)	447 (31)
Mean number of species per ha	207 (12)	201 (12)	84 (8)
Mean Fishers α per ha	40.1 (3.3)	39.4 (3.4)	30.8 (4.6)
Mean basal area per ha	31.65 (4.11)	5.23 (0.55)	26.42 (3.94)
Mean AGB per ha	369.41 (82.30)	18.33 (2.11)	351.08 (81.83)

### Abundances

**Fig 4:**
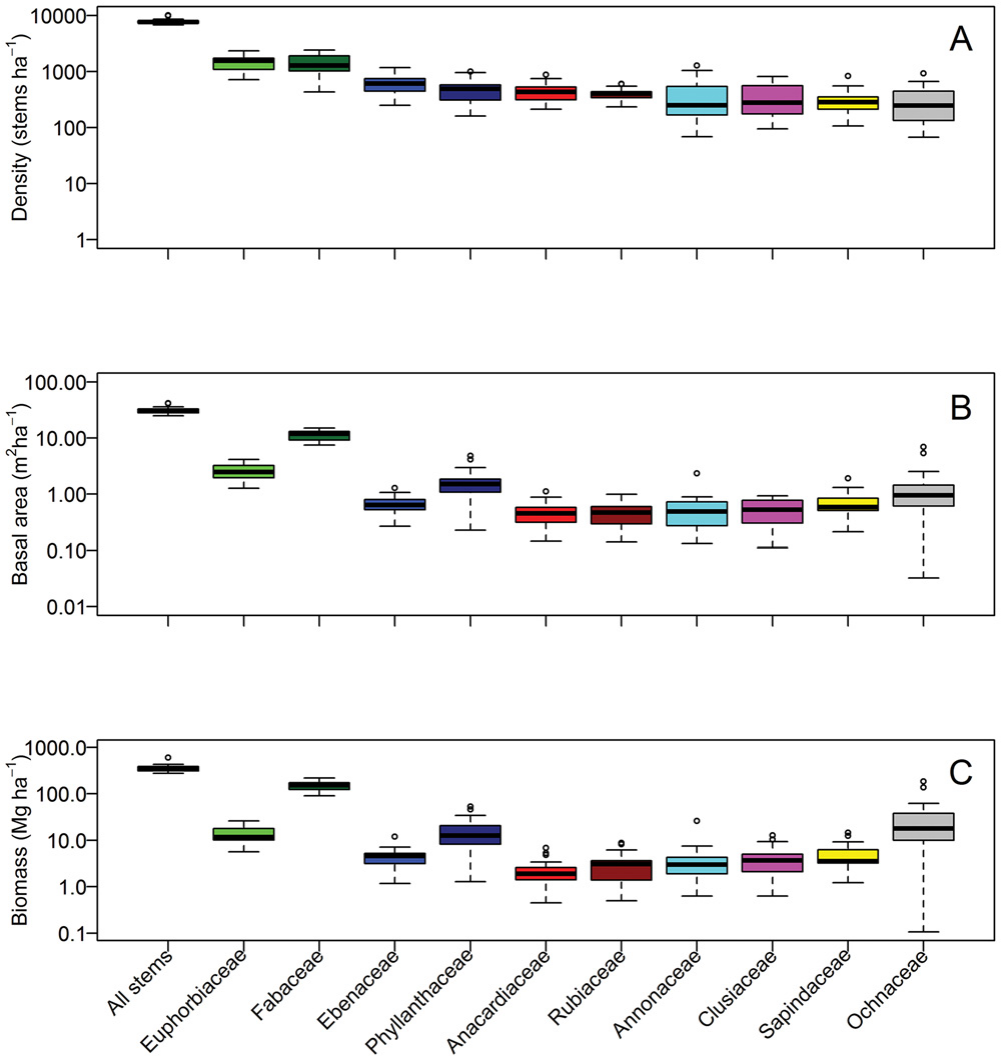
The Density (A), basal area (B), and aboveground live biomass (C) of the ten most abundant tree families in the Rabi plot. Whiskers indicate the 2.5% and 97.5% values of the 25, 100 m × 100 m individual hectares of the plot.

In the 25-ha plot, a total of 175,830 trees were recorded, with an average density of 7,026 individuals/ha. Small-diameter trees were 14 times more abundant than large-diameter trees. There were 164,491 small-diameter trees (93.6% of all stems), averaging 6580 individuals/ha, while large-diameter trees had a density of only 447 individuals/ha (Table 1). The families Euphorbiaceae, and Fabaceae were the most abundant, with densities >1000 individuals/ha, followed by Ebenaceae, Anacardiaceae, Phyllanthaceae, Rubiaceae, and Clusiaceae (Fig 4). Among genera, *Dichostemma* and *Crotonogyne*, both of the family Euphorbiaceae, were the most abundant, with >570 individuals/ha, followed by *Diospyros*, *Garcinia*, *Trichoscypha*, *Tetraberlinia*, *Gilbertiodendron* and *Campylospermum* (Table 2).

**Table 2:** The 20 most abundant tree genera in the 25-ha Rabi plot ranked by density. Basal area and above-ground biomass (AGB) rank indicated in parenthesis.

Genus	Density (trees/ha)	Basal area (m^2^/ha)	AGB (Mg/ha)
*Dichostemma*	683.9	1.4 (2)	5.7 (23)
*Crotonogyne*	573.1	0.2 (42)	0.4 (84)
*Diospyros*	552.4	0.7 (12)	4.5 (26)
*Garcinia*	348.1	0.5 (24)	2.5 (35)
*Trichoscypha*	294.5	0 (186)	1.2 (57)
*Tetraberlinia*	290.5	0.9 (9)	32.3 (1)
*Gilbertiodendron*	266.5	1.6 (1)	18.8 (3)
*Campylospermum*	250.2	0.2 (41)	0.9 (63)
*Pancovia*	175.8	0.5 (26)	2.7 (34)
*Piptostigma*	131.2	0.2 (51)	0.4 (86)
*Warneckea*	126.5	0.1 (179)	1.9 (44)
*Maesobotrya*	123.6	0.2 (45)	0.9 (64)
*Calpocalyx*	122.8	0.4 (30)	2.7 (33)
*Sorindeia*	121.8	0.3 (37)	1.2 (56)
*Protomegabaria*	119.8	0.5 (27)	3.1 (31)
*Dialium*	109.6	0.6 (19)	6.9 (17)
*Diogoa*	100.7	0.6 (20)	5.1 (24)
*Didelotia*	82.1	0.9 (8)	11.3 (7)
*Dactyladenia*	81.1	0.6 (18)	6.6 (21)
*Anisophyllea*	72.4	0.6 (15)	6.8 (20)

At species level, *Dichostemma glaucescens* and *Crotonogyne gabonensis* were the most abundant with densities > 500 individuals/ha, followed by *Garcinia smeathmannii* and *Campylospermum congestum* with densities >200 individuals/ha (Table 3). See S1 Appendix for complete listings of density, basal area and above ground biomass by family, genus, and species.

**Table 3:** The 20 most abundant tree species in the 25-ha Rabi plot ranked by density. Basal area and above-ground biomass (AGB) rank indicated in parenthesis.

Species	Density (trees/ha)	Basal area (m^2^/ha)	AGB (Mg/ha)
*Dichostemma glaucescens*	683.9	1.4 (1)	5.7 (21)
*Crotonogyne gabonensis*	573.1	0.2 (43)	0.4 (107)
*Garcinia smeathmannii*	304.3	0.4 (28)	1.9 (46)
*Campylospermum congestum*	213.8	0.1 (96)	0.7 (80)
*Diospyros obliquifolia*	191.1	0.1 (149)	0.3 (128)
*Pancovia* sp. nov.	175.8	0.5 (23)	2.7 (38)
*Diospyros* sp. nov.	158.3	0.1 (151)	0.2 (145)
*Tetraberlinia moreliana*	149.7	0 (306)	19.8 (2)
*Gilbertiodendron ogoouense*	135.8	0.7 (9)	7.0 (15)
*Piptostigma multinervium*	131.2	0.2 (54)	0.4 (114)
*Tetraberlinia bifoliolata*	123.3	0.9 (6)	9.2 (9)
*Calpocalyx dinklagei*	121.9	0.3 (33)	1.5 (54)
*Protomegabaria stapfiana*	119.7	0.5 (24)	3.1 (36)
*Warneckea floribunda*	109.3	0 (336)	1.4 (56)
*Sorindeia gabonensis*	103.8	0.2 (55)	1.1 (64)
*Diogoa zenkeri*	100.7	0.6 (13)	5.1 (23)
*Gilbertiodendron unijugum*	79.0	0.4 (29)	2.9 (37)
*Diospyros hoyleana*	75.5	0.2 (47)	0.9 (68)
*Trichoscypha* sp.8	69.9	<0.1 (327)	0.2 (158)
*Amanoa strobilacea*	65.5	0.9 (5)	11.1 (6)

**Fig 5:**
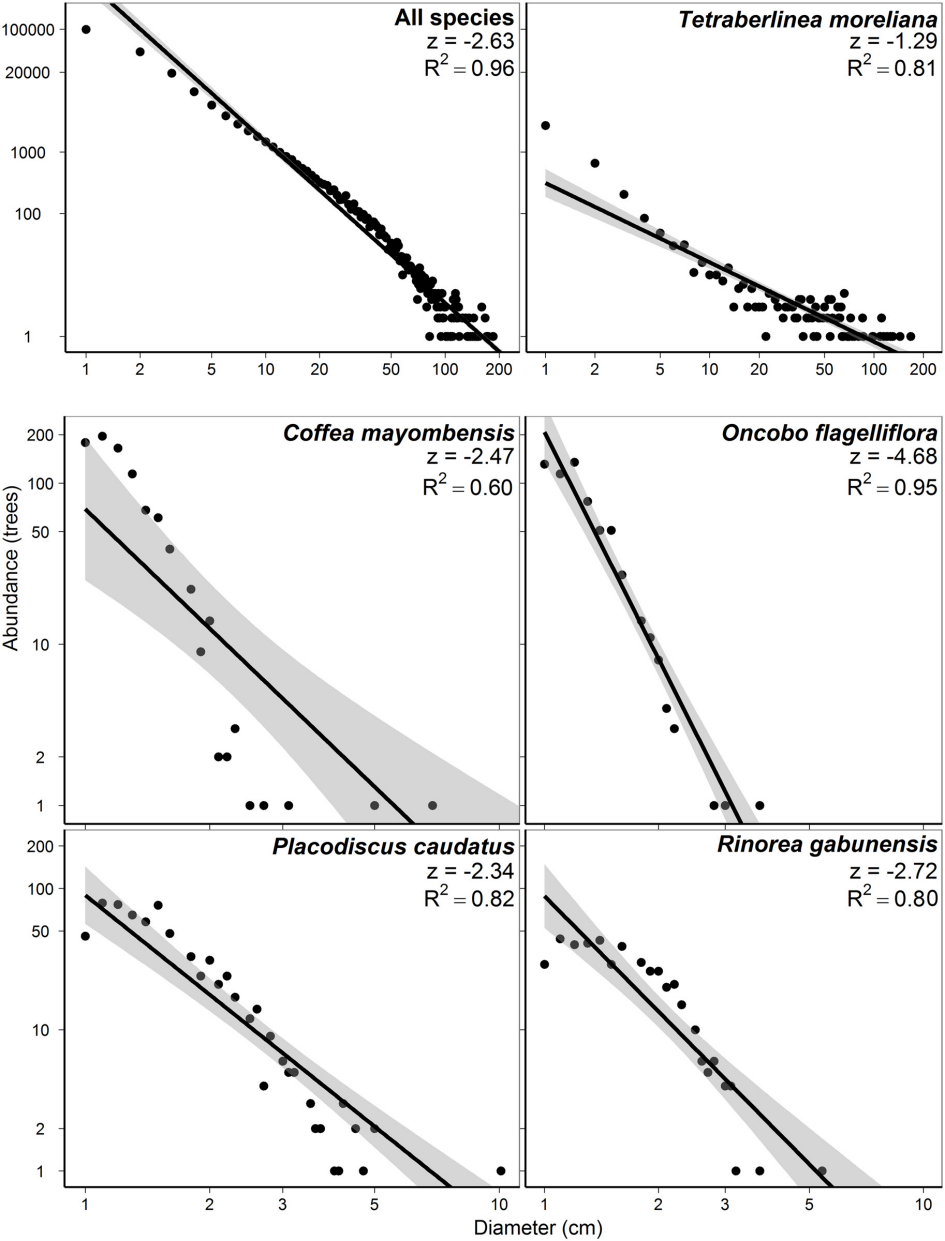
Abundance-diameter relationships for trees within the 25-ha Rabi plot. The relationship between diameter and abundance for all trees (A) has a linear decline in log-log coordinates of z = -2.63 (R^2^ = 0.96). An abundant canopy tree, *Tetraberlinia moreliana* (B), which includes the largest individual tree in the plot, and is well represented in all diameter classes, declines less sharply (z = -1.29, R^2^ = 0.81), and four treelet species, *Coffea mayombensis* (C; R^2^ = 0.60), *Oncoba flagelliflora* (D; R^2^ = 0.95), *Placodiscus caudatus* (E; R^2^ = 0.82), and *Rinorea gabunensis* (F; R^2^ = 0.80), have very steep declines in abundance with increasing diameter. One individual of *Coffea mayombensis* (dbh = 14.9 cm) and three individuals of *Placodiscus caudatus* (dbh = 10.1 cm, 10.2 cm, and 13.4 cm) not shown, but included in calculations. All relationships P <0.001.

Tree abundances declined rapidly with increasing diameter. The forest structure at Rabi follows a declining exponential distribution, with steeper declines at diameters >50 cm dbh (Fig 5). For all species and all diameters, abundance decreased with a slope of -2.63 on a log-log scale (Fig 5) although some species (e.g., *Tetraberlinia moreliana*) declined more gradually. Small-diameter trees, especially treelet lifeforms, declined rapidly within the 1 cm to 10 cm dbh range (Fig 5).

Species with the greatest frequency of occurrence were the understory trees *Garcia smeathmannii* (91% of 625 quadrats) and *Pancovia* sp. (82%); the lower canopy trees *Diogoa zenkeri* (89%), and the treelet *Crotonogyne gabonensis* (84%). The high number of small trees is driven by four treelet species, *Coffea mayombensis* (Rubiaceae), *Oncoba flagelliflora* (Salicaceae), *Rinorea gabunensis* (Violaceae), and *Placodiscus caudatus* (Sapindaceae) (Fig 4). A total of 104 species (30%) had densities of less than one individual per ha, of which 32 species were represented by only one individual in the 25-ha plot.

### Basal Area

Total basal area was 791.23 m^2^ (31.65± 4.11 m^2^/ha) and was dominated by Fabaceae (11.37 m^2^/ha; 35.9% of total), followed by Euphorbiaceae, Phyllanthaceae, Ochnaceae, Simaroubaceae and Burseraceae. The dominant genera by basal area were *Tetraberlinia*, *Gilbertiodendron*, *Odyendea*, *Dischostemma*, *Eurypetalum* and *Lophira*. At species level, *Tetraberlinia moreliana* was the most important, followed by *Odyendyea gabonensis*, *Dichostemma glaucescens*, *Eurypetalum tessmannii*, *Lophira alata* and *Tetraberlinia bifoliolata* (Table 3). Small-diameter trees contributed 16.5% of the total basal area.

### Aboveground biomass

Total aboveground biomass for the 25-ha plot was 9235.18 Mg, averaging 369.41±82.3 Mg/ha for all trees ≥1cm dbh. Fabaceae had the highest AGB (150 Mg/ha), followed by Ochnaceae (33 Mg/ha), Phyllanthaceae (16 Mg/ha), Burseraceae (14 Mg/ha) and Euphorbiaceae (14 Mg/ha). At genus level, *Tetraberlinia* and *Lophira* were the most important (Table 4). *Lophira alata* was the most important species in terms of aboveground biomass, followed by *Tetraberlina moreliana*, *Eurypetalum tessmannii* and *Librevillea klainei*. The aboveground biomass of small-diameter trees was 18 Mg/ha, approximately 5% of the total biomass (Table 4). AGB for large-diameter trees was 351 Mg/ha.

**Table 4:** Comparison of the number of species, abundances, basal area and aboveground biomass (percent of total in parentheses) between small-diameter trees (1 cm ≥ dbh <10 cm) and large-diameter trees with (dbh ≥10cm) per tree life form in the Rabi plot.

	≥ 1 cm	<10 cm	≥ 10 cm
**Species**			
Treelets	105 (30.4%)	105 (30.4%)	14 (4.1%)
Understory	96 (27.8%)	95 (27.5%)	88 (25.5%)
Lower canopy	83 (24.1%)	79 (22.9%)	78 (22.6%)
Upper canopy	61 (17.7%)	54 (15.7%)	54 (15.7%)
**Totals**	**345**	**333 (96.5%)**	**234 (67.8%)**
**Abundance**			
Treelets	38,474 (22.1%)	38,413 (22.1%)	61 (<0.1%)
Understory	55,605 (32.0%)	53,084 (30.6%)	2,520 (1.5%)
Lower canopy	53,331 (30.7%)	48,558 (28.0%)	4,769 (2.8%)
Upper canopy	26,311 (15.1%)	22,632 (13.0%)	3,674 (2.1%)
**Totals**	**173,721**	**162,687 (93.6%)**	**11,024 (6.4%)**
**Basal Area (m**^**2**^**)**			
Treelets	15.63 (2.0%)	14.8 (1.9%)	0.83 (0.1%)
Understory	91.42 (11.7%)	44.98 (5.8%)	46.44 (5.9%)
Lower canopy	221.99 (28.4%)	46.29 (5.9%)	175.7 (22.5%)
Upper canopy	452.83 (57.9%)	23.28 (3.0%)	429.56 (55.0%)
**Totals**	**781.87**	**129.35 (16.6%)**	**652.53 (83.5%)**
**Aboveground Biomass (Mg)**			
Treelets	55.39 (0.6%)	50.26 (0.6%)	5.13 (0.1%)
Understory	549.14 (6.0%)	171.04 (1.9%)	378.1 (4.1%)
Lower canopy	2,019.66 (22.1%)	154.02 (1.7%)	1,865.64 (20.4%)
Upper canopy	6,512.31 (71.3%)	89.71 (1.0%)	6,422.60 (70.3%)
**Totals**	**9,136.50**	**465.03 (5.1%)**	**8,671.47 (94.9%)**

### Forest structure by lifeform

Among the 345 species in the Rabi plot, there were 105 treelet species, with diameters mostly restricted to <10 cm. These accounted for 22% of the total number of individuals in the plot, but only 2% of basal area and 0.6% of aboveground biomass respectively (Table 4). Three of the 20 most abundant species in the plot were treelets, *Crotonogyne gabonensis*, *Campylospermum congestum* and *Diospyros* sp. nov. (Table 4). The remaining 240 species comprised 96 understory, 83 lower canopy and 61 upper canopy species, all of which reach more than 10 m in height and dbh ≥10 cm at maturity. These species represented 78% of the total number of trees, 98% of the total basal area and 99% of the total aboveground biomass.

Small-diameter trees comprised all 105 species of treelets, but also 95%, 91% and 86% of understory, lower canopy and upper canopy species respectively. Treelets accounted for 23.6% of the total individuals, 11.5% of the basal area and 10.8% of the aboveground biomass in this diameter size class. Understory and canopy species had at least 88% of their species among these small-diameter trees, and accounted for 78.4% of all trees, 85% of the basal area and 89% of the aboveground biomass of small-diameter trees. Among trees with dbh ≥10 cm, there were only 14 species of treelets, representing a tiny fraction of their total individuals, total basal area and of the aboveground biomass in this diameter class. Conversely upper canopy species with 33% of the individual accounted for 66% of the basal area and 74% of the aboveground biomass in this diameter class (Table 4).

## Discussion

Although there have been some concerted effort in the recent past towards long-term monitoring of tropical African forests (e.g., the African Tropical Rainforest Observation Network; http://www.afritron.org), studies that include small-diameter trees are still uncommon, especially in the Congo Basin. The 25-ha Rabi plot is only the third large continuous patch of forest in Africa within which all trees with dbh ≥1cm are censused. Our results show clear differences in species diversity, abundances, basal area and aboveground biomass between small- and large-diameter trees. As in Korup and Ituri forests [13–15], lowering the sampling diameter to 1 cm in the Rabi plot increased the interpretation of the total diversity and the density of species.

Small-diameter trees were more diverse than large-diameter trees in the Rabi plot based on Fisher’s α. At least 30% of the species in the plot were treelets that achieve reproductive maturity in the forest understory and never attain 10 cm dbh. In addition to these treelets, small-diameter trees comprised saplings of all other understory and canopy tree species that do regenerate.

Tree density in the Rabi plot was comparable to other tropical African and temperate forests (Table 5). The African plots have generally higher proportions (92% to 95%) of small-diameter trees compared to other tropical plots, but the plot at Sinharaja, Sri Lanka was comparable. Temperate plots or dry tropical plots have much lower proportions (30% to 70%) of small-diameter trees, perhaps because of the combination of climatic limitations and repeated low intensity disturbance from fire or herbivory. The Rabi plot was notable in the steepness of the decline in the abundance-diameter relationship (Fig 5, Table 5). When only large-diameter trees are considered, the Rabi forest is dominated by Fabaceae, Olacaceae and Euphorbiaceae, which is in agreement with previous forest inventories in the area that used smaller plots (0.1 ha) with a 5 cm minimum diameter [21,23]. As in the Korup plot in Cameroon, *Dichostemma glaucescens* was the most abundant canopy species [14]. The abundance of small-diameter trees in the Rabi forest is not unique. Indeed, the high density of small trees has been reported in the Congo Basin [13,14] and in other tropical forests worldwide [16,30–32], and stands in contrast to densities in temperate plots (e.g., [33,34]). For example, within the Rabi plot, small-diameter trees account for 93.6% of all trees while in the 50-ha plot in Korup, they make up 92.4%. Small-diameter trees in Rabi were predominantly composed of saplings of understory and canopy tree species (76.4% of all trees in this size class). Saplings are more vulnerable to environmental fluctuations as well as damaging agents such as browsing herbivores and being crushed by windfalls. Long-term monitoring of this life stage is crucial to understanding the demography of canopy species as well as possible changes to forest composition driven by global change [12].

**Table 5:** Comparison of the Rabi, Gabon plot to other African CTFS plots and to tropical plots in South America and Asia, as well as two temperate plots in the USA. African CTFS plots have a higher proportion of small-diameter trees than other tropical plots, and many more than temperate plots. Z represents the negative exponent of the abundance-diameter relationship (see Methods).

CTFS-ForestGEO Site	Country	Area (ha)	Census year	Trees dbh≥ 1cm (Ind./ha)	Trees dbh<10 cm (Ind./ha)	Trees dbh≥ 10 cm (Ind./ha)	Trees dbh<10 cm (%)	Z
Rabi	Gabon	25	2013	7,026.4	6,579.7	446.8	93.6	2.6
Ituri-Lenda	DR Congo	20	1995	6,843.6	6,486.0	357.6	94.8	2.1
Ituri-Edoro	DR Congo	20	1995	8,112.1	7,673.5	438.6	94.6	2.1
Sinharaja	Sri Lanka	25	1995	8,215.0	7,537.5	677.5	91.8	2.1
Korup	Cameroon	50	1999	6,580.6	6,070.7	509.9	92.3	2.0
Lambir	Malaysia	52	1997	6,915.5	6,277.5	638.0	90.8	2.0
BCI	Panama	50	2000	4,276.1	3,852.0	424.1	90.1	1.9
Pasoh	Malaysia	50	2000	6,118.9	5,553.3	565.6	90.8	1.9
Yasuni	Ecuador	25	1997	6,094.2	5,392.3	701.9	88.5	1.9
Mudumalai	India	50	2000	360.5	109.0	251.6	30.2	1.2
Yosemite	USA	25.6	2010	1,346.1	818.1	528.0	60.8	2.0
Wind River	USA	25.6	2011	1,209.9	884.7	325.3	73.1	1.8

That forest basal area and biomass is disproportionately carried in the large-diameter trees could make the forest at Rabi less resilient to short-term environmental change or disturbance. If the large-diameter individuals were killed, either through disturbance, disease, or human agency, the higher Z, relative to other forests (Table 5) suggests that it could take a relatively longer time for smaller-diameter trees to advance to the overstory. However, the diversity and abundance of the small-diameter trees suggests that the forest could have the ability to respond to disturbances in general. The ability of the forest to recover from disturbances affecting small-diameter trees through the regeneration of small-diameter trees suggests a high level of structural resilience—the perpetration of the existing diameter distribution within the forest. Small-diameter trees. Aboveground biomass in Rabi averaged 351 Mg/ha for trees ≥ 1cm which is lower than the estimated African mean of 395.7 Mg [32]. This lower value is probably due to the prevalence of small-diameter trees in this forest, and the limited selective logging of large-diameter trees that occurred two decades previously (site examination suggests that only the 11 large trees were removed). Small-diameter trees in the Rabi plot stored 5.0% of the total biomass while in the Korup plot, they accounted for 5.7% of the total biomass. This result is in agreement with the assumption that in mature tropical forests, the biomass of small-diameter trees is approximately 5% of the total aboveground biomass [25,33,34]. Our study also confirms that large-diameter trees store the bulk of biomass in tropical forests. In Rabi, 95% of the biomass is stored in trees with dbh ≥ 10 cm. Within this diameter class, upper canopy trees, comprised only 2% of the total individual trees in the plot, but stored 70% of the total biomass. Therefore, the contribution of small-diameter trees to biomass may be approximated based on forest type averages and forest AGB can be estimated from the few large trees [4,33], thereby avoiding the labor intensive work of censusing the small-diameter trees. However the demography of small-diameter trees is important to predicting the long-term change in above-ground biomass of the forest. For example, in subtropical evergreen broad leaved forests of China, Lin et al. [17] showed that small-diameter trees contributed 10.4% of the total above ground biomass, more than trees ≥50 cm dbh. Therefore the dynamics of the small-diameter trees in this forest could be relatively more important to overall ecosystem function—an opposite conclusion from many studies emphasizing large-diameter trees (e.g., [4,30,34]).

As the Congo Basin forest is considered to be the second most important forested region on earth after the Amazon, it is crucial that more comprehensive long-term studies including small diameter trees be implemented throughout its range.

## Supporting Information

S1 Appendix Abundance, basal area, and aboveground biomass (AGB) of woody species ≥1 cm dbh in the 25-ha Rabi forest plot, Gabon. (DOCX)
