# Supplementary material for: Ecological Importance of Small-Diameter Trees to the Structure, Diversity and Biomass of a Tropical Evergreen Forest at Rabi, Gabon
Source: PLoS One. 2016 May 17;11(5):e0154988. doi: 10.1371/journal.pone.0154988 (PMC4871370; doi:10.1371/journal.pone.0154988)
Supplement: S1 Appendix — (DOCX) [file pone.0154988.s001.docx]

Supplemental Information

Appendix S1. Abundance, basal area, and aboveground biomass (AGB) of woody species ≥1 cm dbh in the 25 ha Rabi forest plot, Gabon

| **Code** | **Family** | **Species** | **N** | **BA (m2)** | **AGB (Mg)** |
| --- | --- | --- | --- | --- | --- |
| THOMCO | Acanthaceae | Thomandersia hensii | 316 | 0.7 | 5 |
| SCOTKL | Achariaceae | Scottellia klaineana | 70 | 0.3 | 1.5 |
| SORIGA | Anacardiaceae | Sorindeia gabonensis | 2,598 | 7.5 | 58.2 |
| SORI02 | Anacardiaceae | Sorindeia juglandifolia | 450 | 1.2 | 7.1 |
| TRICAC | Anacardiaceae | Trichoscypha acuminata | 135 | 0.3 | 1.3 |
| TRICPE | Anacardiaceae | Trichoscypha lucens | 1,491 | 30.5 | 414.2 |
| TRICLP | Anacardiaceae | Trichoscypha reygaertii | 1 | 0.1 | 0.1 |
| TRICGL | Anacardiaceae | Trichoscypha rubicunda | 478 | 3.2 | 43.8 |
| TRICCM | Anacardiaceae | Trichoscypha soyauxii | 994 | 0.3 | 0.7 |
| TRIC02 | Anacardiaceae | Trichoscypha sp.1 | 27 | 0.1 | 0.2 |
| TRIC03 | Anacardiaceae | Trichoscypha sp.2 | 246 | 0.6 | 4.1 |
| TRICCD | Anacardiaceae | Trichoscypha sp.3 | 570 | 0.5 | 2.6 |
| TRICFL | Anacardiaceae | Trichoscypha sp.4 | 1,018 | 17.5 | 328.8 |
| TRICFO | Anacardiaceae | Trichoscypha sp.5 | 239 | 0.1 | 0.3 |
| TRICIN | Anacardiaceae | Trichoscypha sp.6 | 376 | 9.7 | 122.9 |
| TRICNO | Anacardiaceae | Trichoscypha sp.7 | 43 | 1 | 11.2 |
| TRICPU | Anacardiaceae | Trichoscypha sp.8 | 1,747 | 3.2 | 17.8 |
| TRICSP | Anacardiaceae | Trichoscypha sp.9 | 1 | 0.1 | 0.1 |
| ANISSP | Anisophylleaceae | Anisophyllea ferruginea | 280 | 2.1 | 24.8 |
| ANISGL | Anisophylleaceae | Anisophyllea polyneura | 860 | 6.7 | 83.1 |
| ANISPU | Anisophylleaceae | Anisophyllea purpurascens | 342 | 3.6 | 44.5 |
| ANIS03 | Anisophylleaceae | Anisophyllea sp. | 328 | 0.6 | 3.9 |
| ANNICH | Annonaceae | Annickia chlorantha | 1 | 0.1 | 0.1 |
| CLEIPA | Annonaceae | Cleistopholis patens | 4 | 0.1 | 0.1 |
| CLEIST | Annonaceae | Cleistopholis staudtii | 3 | 0.1 | 0.1 |
| DUGUCO | Annonaceae | Duguetia confinis | 245 | 0.2 | 0.4 |
| GREESU | Annonaceae | Greenwayodendron suaveolens | 744 | 0.8 | 4.3 |
| GREEGA | Annonaceae | Greenwayodendron suaveolens var. gabonica | 611 | 0.6 | 3 |
| ISOLHE | Annonaceae | Isolona hexaloba | 247 | 0.4 | 2.5 |
| PIPTMU | Annonaceae | Piptostigma sp. nov. | 3,290 | 8.7 | 64.9 |
| POLYPA | Annonaceae | Polyceratocarpus parviflorus | 150 | 0.1 | 0.2 |
| UVAR02 | Annonaceae | Uvariastrum pierreanum | 23 | 0.1 | 0.1 |
| XYLOAE | Annonaceae | Xylopia aethiopica | 165 | 1.4 | 11.2 |
| XYLO02 | Annonaceae | Xylopia le-testui | 190 | 1.1 | 8.5 |
| XYLOLF | Annonaceae | Xylopia parviflora | 101 | 0.5 | 3.6 |
| XYLOPF | Annonaceae | Xylopia pynaertii | 544 | 3.5 | 28.8 |
| XYLOQU | Annonaceae | Xylopia quintasii | 120 | 0.8 | 5.3 |
| ANNO02 | Annonaceae | Xylopia sp.1 | 473 | 1.6 | 17.1 |
| ANNO03 | Annonaceae | Xylopia sp.2 | 4 | 0.1 | 0.1 |
| APOC02 | Apocynaceae | Malouetia mildbraedii | 40 | 0.1 | 0.3 |
| PICRNI | Apocynaceae | Pleiocarpa brevistyla | 10 | 0.2 | 1.5 |
| RAUVVO | Apocynaceae | Rauvolfia vomitoria | 2 | 0.1 | 0.1 |
| VOAC02 | Apocynaceae | Voacanga bracteata | 18 | 0.1 | 1 |
| AUCOKL | Burceraceae | Aucoumea klaineana | 8 | 0.1 | 0.1 |
| DACRBU | Burceraceae | Dacryodes buettneri | 8 | 0.1 | 0.1 |
| DACRED | Burceraceae | Dacryodes edulis | 90 | 0.1 | 0.1 |
| DACRIG | Burceraceae | Dacryodes igaganga | 268 | 0.1 | 0.2 |
| DACRKL | Burceraceae | Dacryodes klaineana | 6 | 0.1 | 0.1 |
| DACRNO | Burceraceae | Dacryodes normandii | 1 | 0.1 | 0.1 |
| SANTTR | Burceraceae | Santiria trimera | 74 | 0.3 | 1.7 |
| CENTGL | Centroplacaceae | Centroplacus glaucinus | 256 | 0.1 | 0.3 |
| DACTPU | Chrysobalanaceae | Dactyladenia bellayana | 209 | 0.1 | 0.2 |
| DACTGL | Chrysobalanaceae | Dactyladenia campestris | 107 | 0.1 | 0.1 |
| DACTPF | Chrysobalanaceae | Dactyladenia letestui | 1,551 | 0.4 | 0.8 |
| DACT02 | Chrysobalanaceae | Dactyladenia librevillensis | 146 | 0.1 | 0.1 |
| DICH03 | Chrysobalanaceae | Dactyladenia staudtii | 15 | 0.6 | 5.8 |
| MAGNBI | Chrysobalanaceae | Magnistipula bimarsupiata | 365 | 1.7 | 17.7 |
| MAGNPU | Chrysobalanaceae | Magnistipula multinervia | 2 | 0.1 | 0.1 |
| MAGN02 | Chrysobalanaceae | Magnistipula sp. | 316 | 0.8 | 5.4 |
| MARACH | Chrysobalanaceae | Maranthes chrysophylla | 9 | 0.1 | 0.1 |
| GARCTA | Clusiaceae | Garcinia chromocarpa | 1,094 | 7.8 | 96.8 |
| GARCKO | Clusiaceae | Garcinia kola | 1 | 0.1 | 0.1 |
| GARCSM | Clusiaceae | Garcinia smeathmannii | 7,612 | 19.9 | 194.2 |
| HARUMA | Clusiaceae | Harungana madagascariensis | 71 | 0.1 | 0.3 |
| SYMPGL | Clusiaceae | Symphonia globulifera | 118 | 0.1 | 0.2 |
| STREMA | Combretaceae | Strephonema mannii | 1,136 | 0.6 | 1.6 |
| STRE02 | Combretaceae | Strephonema sericeum | 372 | 0.2 | 0.5 |
| HEMA03 | Connaraceae | Hemandradenia chevalieri | 1 | 0.1 | 0.1 |
| HEMA02 | Connaraceae | Hemandradenia mannii | 190 | 0.2 | 0.8 |
| JOLL02 | Connaraceae | Jollydora pierrei | 40 | 0.1 | 0.3 |
| COULED | Coulaceae | Coula edulis | 646 | 2.9 | 25.3 |
| DICH02 | Dichapetalaceae | Dichapelum sp. | 3 | 0.3 | 2.3 |
| DIOSCC | Ebenaceae | Diospyros boala | 159 | 0.4 | 1.3 |
| DIOSCI | Ebenaceae | Diospyros cinnabarina | 1,330 | 2.3 | 9.1 |
| DIOSCO | Ebenaceae | Diospyros conocarpa | 6 | 0.1 | 0.1 |
| DIOSDE | Ebenaceae | Diospyros deltoidea | 133 | 0.3 | 1.3 |
| DIOSFR | Ebenaceae | Diospyros fragrans | 537 | 1.7 | 14 |
| DIOSHO | Ebenaceae | Diospyros hoyleana | 1,887 | 19.5 | 234.6 |
| DIOSMA | Ebenaceae | Diospyros mannii | 29 | 0.1 | 0.3 |
| DIOSOB | Ebenaceae | Diospyros obliquifolia | 4,787 | 5.7 | 29.9 |
| DIOSPI | Ebenaceae | Diospyros piscatoria | 277 | 0.2 | 0.4 |
| DIOSRA | Ebenaceae | Diospyros rabiensis | 366 | 0.2 | 0.4 |
| DIOSIT | Ebenaceae | Diospyros sp. nov. | 3,958 | 15.4 | 126.7 |
| DIOSBO | Ebenaceae | Diospyros vermoesenii | 356 | 0.7 | 2.6 |
| CROT02 | Euphorbiaceae | Crotonogyne gabonensis | 14,336 | 15 | 173.4 |
| DICHGL | Euphorbiaceae | Dichostemma glaucescens | 17,108 | 55.8 | 501.2 |
| DISCCA | Euphorbiaceae | Discoglypremna caloneura | 6 | 0.1 | 0.1 |
| KLAIGA | Euphorbiaceae | Klaineanthus gaboniae | 646 | 0.8 | 4.3 |
| MACAMO | Euphorbiaceae | Macaranga monandra | 5 | 0.1 | 0.1 |
| MACASP | Euphorbiaceae | Macaranga spinosa | 6 | 0.1 | 0.1 |
| MAPRME | Euphorbiaceae | Maprounea membranacea | 342 | 1.5 | 18.5 |
| MARE02 | Euphorbiaceae | Mareyopsis longifolia | 393 | 1.4 | 12.4 |
| PLAGAF | Euphorbiaceae | Plagiostyles africana | 118 | 0.1 | 0.2 |
| AFZEBI | Fabaceae | Afzelia bipindensis | 8 | 0.1 | 0.1 |
| ALBIGL | Fabaceae | Albizia glaberrima | 32 | 0.1 | 0.1 |
| ANGY02 | Fabaceae | Angylocalyx cf. oligophyllus | 742 | 4.4 | 56.7 |
| ANGY03 | Fabaceae | Angylocalyx sp. | 16 | 0.1 | 0.1 |
| ANTHCP | Fabaceae | Anthonotha crassifolia | 349 | 3.4 | 18.1 |
| BAIK02 | Fabaceae | Baikiaea insignis | 55 | 0.1 | 0.3 |
| BAPH02 | Fabaceae | Baphia hylophila | 1,446 | 9.5 | 117.4 |
| BAPHGF | Fabaceae | Baphia sp. | 245 | 1.2 | 12.8 |
| BERL02 | Fabaceae | Berlinia confusa | 889 | 3.5 | 28.8 |
| BERLCB | Fabaceae | Berlinia rabiensis | 70 | 0.4 | 2.4 |
| BIKI02 | Fabaceae | Bikinia pellegrinii | 41 | 0.4 | 3.1 |
| CALPDI | Fabaceae | Calpocalyx dinklagei | 3,050 | 26 | 344.3 |
| CALPHE | Fabaceae | Calpocalyx heitzii | 21 | 0.1 | 0.2 |
| COPAMI | Fabaceae | Copaifera mildbraedii | 3 | 0.1 | 0.1 |
| CRUD02 | Fabaceae | Crudia gabonensis | 742 | 0.3 | 0.6 |
| CRYPPE | Fabaceae | Cryptosepalum pellegrinianum | 343 | 0.1 | 0.2 |
| DIALAN | Fabaceae | Dialium angolense | 1,364 | 8.6 | 130.3 |
| DIALCO | Fabaceae | Dialium corbisieri | 2 | 0.1 | 0.3 |
| DIALDI | Fabaceae | Dialium dinklagei | 1 | 0.1 | 0.1 |
| DIALPF | Fabaceae | Dialium guineense | 1,095 | 8.1 | 128.9 |
| DIALTE | Fabaceae | Dialium tessmannii | 278 | 13.7 | 172.3 |
| DIDEAF | Fabaceae | Didelotia africana | 1,462 | 2.6 | 10.5 |
| DIDEBR | Fabaceae | Didelotia brevipaniculata | 127 | 0.3 | 1 |
| DIDEMI | Fabaceae | Didelotia minutiflora | 76 | 0.1 | 0.4 |
| CRYP02 | Fabaceae | Didelotia unifoliolata | 389 | 0.1 | 0.2 |
| EURYTE | Fabaceae | Eurypetalum tessmannii | 566 | 0.2 | 0.6 |
| HYMENG | Fabaceae | Gabonius ngouniensis | 539 | 0.5 | 2.8 |
| GILBOG | Fabaceae | Gilbertiodendron ogoouense | 3,399 | 4.7 | 36.9 |
| GILB02 | Fabaceae | Gilbertiodendron sp. | 753 | 7.1 | 78 |
| GILBST | Fabaceae | Gilbertiodendron stipulaceum | 544 | 0.6 | 3.3 |
| GILBCU | Fabaceae | Gilbertiodendron unijugum | 1,976 | 23.1 | 274.3 |
| GUIBEH | Fabaceae | Guibourtia ehie | 1 | 0.1 | 0.1 |
| HYMEFL | Fabaceae | Hymenostegia floribunda | 1,067 | 1.3 | 8.1 |
| HYMEKL | Fabaceae | Hymenostegia klainei | 5 | 0.1 | 0.1 |
| LIBRKL | Fabaceae | Librevillea klainei | 939 | 9.9 | 156 |
| NEOCST | Fabaceae | Neochevalierodendron stephanii | 845 | 3.9 | 54.4 |
| NEWTLE | Fabaceae | Newtonia leucocarpa | 8 | 0.2 | 2.3 |
| PARKBI | Fabaceae | Parkia bicolor | 1 | 0.1 | 0.1 |
| PTERSO | Fabaceae | Pterocarpus soyauxii | 1 | 0.1 | 0.1 |
| SINDKL | Fabaceae | Sindora klaineana | 173 | 0.4 | 1.8 |
| TETRBI | Fabaceae | Tetraberlinia bifoliolata | 3,083 | 6.4 | 60.3 |
| TETRLO | Fabaceae | Tetraberlinia longiracemosa | 440 | 1.5 | 10.4 |
| TETRMO | Fabaceae | Tetraberlinia moreliana | 3,742 | 9 | 59.9 |
| ANTHVO | Gentianaceae | Anthocleista vogelii | 158 | 2.8 | 17.4 |
| ANTHAU | Gentianaceae | Anthostema aubryanum | 1,059 | 9 | 63.7 |
| AFROKA | Huaceae | Afrostyrax kamerunensis | 1,278 | 0.5 | 1.4 |
| SACOGA | Humiriaceae | Sacoglottis gabonensis | 97 | 0.2 | 1.1 |
| DESM02 | Icacinaceae | Desmostachys oblongifolius | 1 | 0.1 | 0.1 |
| DESBIN | Irvingiaceae | Desbordesia insignis | 1 | 0.1 | 0.1 |
| IRVIGA | Irvingiaceae | Irvingia gabonensis | 84 | 0.1 | 0.5 |
| IRVIGR | Irvingiaceae | Irvingia grandifolia | 3 | 0.1 | 0.1 |
| IRVIRO | Irvingiaceae | Irvingia robur | 1 | 0.1 | 0.1 |
| KLAI02 | Irvingiaceae | Klainedoxa gabonensis | 17 | 0.1 | 0.2 |
| OCHTAF | Ixonanthaceae | Ochthocosmus africanus | 4 | 0.1 | 0.4 |
| OCHTCA | Ixonanthaceae | Ochthocosmus calothyrsus | 925 | 11.7 | 193.3 |
| VITEDO | Lamiaceae | Vitex doniana | 65 | 0.1 | 0.6 |
| VITE02 | Lamiaceae | Vitex gabunensis | 27 | 0.1 | 0.9 |
| BEILNV | Lauraceae | Beilschmiedia auriculata | 384 | 1.7 | 13.9 |
| BEILPU | Lauraceae | Beilschmiedia fulva | 88 | 0.3 | 1.6 |
| BEIL02 | Lauraceae | Beilschmiedia gaboonensis | 602 | 1.8 | 13.8 |
| BEILGF | Lauraceae | Beilschmiedia pierreana | 266 | 0.7 | 6.5 |
| BEIL03 | Lauraceae | Beilschmiedia sp.1 | 230 | 0.8 | 7.4 |
| BEILLF | Lauraceae | Beilschmiedia sp.2 | 133 | 0.7 | 5.1 |
| BEILPF | Lauraceae | Beilschmiedia sp.3 | 32 | 0.1 | 0.7 |
| BEIL04 | Lauraceae | Beilschmiedia sp.4 | 5 | 0.1 | 0.4 |
| CRAT02 | Lecythidaceae | Crateranthus sp. nov. | 969 | 1.7 | 16.2 |
| NAPOGF | Lecythidaceae | Napoleonaea imperialis | 378 | 3.8 | 33.9 |
| NAPO02 | Lecythidaceae | Napoleonaea vogelii | 718 | 0.4 | 2 |
| OUBAAF | Lecythidaceae | Oubanguia africana | 796 | 7.7 | 118.9 |
| SCYTFL | Lecythidaceae | Oubanguia laurifolia | 56 | 0.2 | 0.9 |
| RHAP02 | Lecythidaceae | Rhaptopetalum sp. nov. | 299 | 0.8 | 6.2 |
| SCYTKL | Lecythidaceae | Scytopetalum klaineanum | 950 | 2.1 | 12 |
| COLAFD | Malvaceae | Chlamydocola lastoursvillensis | 655 | 2 | 14 |
| COLALA | Malvaceae | Cola lateritia | 52 | 0.1 | 0.6 |
| COLA03 | Malvaceae | Cola sp. | 1 | 0.1 | 0.1 |
| GREW02 | Malvaceae | Grewia oligoneura | 22 | 0.1 | 0.3 |
| LEPT02 | Malvaceae | Leptonychia sp. | 1 | 0.1 | 0.1 |
| GREWCO | Malvaceae | Microcos coriacea | 88 | 0.1 | 0.6 |
| NESO02 | Malvaceae | Nesogordonia kabingaensis | 90 | 0.5 | 6.7 |
| COLA02 | Malvaceae | Octolobus spectabilis | 148 | 0.4 | 2.6 |
| PTERBE | Malvaceae | Pterygota bequaertii | 12 | 0.1 | 0.1 |
| DICHAF | Melastomataceae | Dichaetanthera africana | 7 | 0.7 | 8.7 |
| MEMETO | Melastomataceae | Memecylon klaineanum | 566 | 0.8 | 5.6 |
| MEMEMO | Melastomataceae | Memecylon nodosum | 53 | 0.2 | 1.2 |
| MEMENV | Melastomataceae | Memecylon sp. nov. | 377 | 1.7 | 21.5 |
| MEME02 | Melastomataceae | Memecylon sp.1 | 331 | 2.2 | 27.4 |
| MEME03 | Melastomataceae | Memecylon sp.2 | 26 | 0.3 | 4.1 |
| MEMEFC | Melastomataceae | Memecylon sp.3 | 193 | 1 | 9.3 |
| MEMEC2 | Melastomataceae | Memecylon viride | 118 | 0.3 | 1.8 |
| SPATBL | Melastomataceae | Spathandra blakeoides | 205 | 0.1 | 0.3 |
| MEMEPF | Melastomataceae | Warneckea bebaiensis | 137 | 0.3 | 1.4 |
| WARN02 | Melastomataceae | Warneckea floribunda | 2,731 | 20.6 | 238 |
| WARN03 | Melastomataceae | Warneckea fosteri | 2 | 0.1 | 0.1 |
| WARNPU | Melastomataceae | Warneckea pulcherrima | 214 | 2 | 17.8 |
| WARNDD | Melastomataceae | Warneckea sp. | 78 | 0.9 | 8.4 |
| TRIICM | Meliaceae | Trichilia gilgiana | 3 | 0.1 | 0.1 |
| TRIITE | Meliaceae | Trichilia tessmannii | 1 | 0.1 | 0.1 |
| TRECAF | Moraceae | Treculia africana | 1 | 0.1 | 0.1 |
| COELPR | Myristicaceae | Coelocaryon preussii | 6 | 0.1 | 0.1 |
| SCYPMA | Myristicaceae | Scyphocephalium mannii | 28 | 0.1 | 0.2 |
| STAUKA | Myristicaceae | Staudtia gabonensis | 111 | 0.1 | 0.2 |
| ARDI02 | Myrsinaceae | Ardisia buesgenii | 42 | 0.1 | 0.6 |
| EUGELF | Myrtaceae | Eugenia klaineana | 213 | 0.1 | 0.2 |
| EUGE02 | Myrtaceae | Eugenia sp. | 215 | 0.1 | 0.2 |
| SYZY03 | Myrtaceae | Syzygium congolense | 6 | 0.1 | 0.1 |
| SYZY02 | Myrtaceae | Syzygium staudtii | 54 | 0.1 | 0.1 |
| CAMP03 | Ochnaceae | Campylospermum calanthum | 21 | 0.1 | 0.1 |
| CAMPDU | Ochnaceae | Campylospermum duparquetianum | 48 | 0.1 | 0.1 |
| CAMPBO | Ochnaceae | Campylospermum engama | 94 | 0.1 | 0.1 |
| CAMPLF | Ochnaceae | Campylospermum glaucum | 455 | 0.2 | 0.6 |
| CAMPFM | Ochnaceae | Campylospermum laxiflorum | 296 | 0.2 | 11.5 |
| CAMP02 | Ochnaceae | Campylospermum sulcatum | 5,345 | 5.9 | 50.6 |
| LOPHAL | Ochnaceae | Lophira alata | 118 | 1.9 | 27.6 |
| OCHN02 | Ochnaceae | Ochna sp. | 3 | 0.1 | 0.1 |
| RHAB02 | Ochnaceae | Rhabdophyllum arnoldianum | 769 | 1.3 | 9.6 |
| OCTOCA | Octoknemaceae | Octoknema affinis | 10 | 0.3 | 3.3 |
| DIOGZE | Olacaceae | Diogoa zenkeri | 2,517 | 4.1 | 16.3 |
| STRO02 | Olacaceae | Strombosia grandifolia | 79 | 0.1 | 0.1 |
| STROPU | Olacaceae | Strombosia pustulata var. pustulata | 1,137 | 0.7 | 2.9 |
| STROTE | Olacaceae | Strombosiopsis tetrandra | 652 | 0.4 | 0.8 |
| MICRPU | Pandaceae | Microdesmis cf. haumaniana | 338 | 0.5 | 3.1 |
| MICRGL | Pandaceae | Microdesmis sp. | 5 | 0.1 | 0.1 |
| BARTFI | Passifloraceae | Barteria fistulosa | 5 | 0.1 | 0.3 |
| PARO02 | Passifloraceae | Paropsia gabonica | 31 | 0.1 | 0.1 |
| PARODE | Passifloraceae | Paropsiopsis decandra | 4 | 0.1 | 0.1 |
| SOYALF | Peridiscaceae | Soyauxia floribunda | 970 | 0.5 | 1.1 |
| AMANST | Phyllanthaceae | Amanoa strobilacea | 1,637 | 19.1 | 255.8 |
| ANTIVO | Phyllanthaceae | Antidesma vogelianum | 288 | 2.4 | 14.7 |
| CLEIIT | Phyllanthaceae | Cleistanthus gabonensis | 120 | 0.1 | 0.1 |
| CLEILE | Phyllanthaceae | Cleistanthus letouzeyi | 189 | 0.1 | 0.4 |
| MAESFA | Phyllanthaceae | Maesobotrya barteri | 1,274 | 5.1 | 46.1 |
| MAESBC | Phyllanthaceae | Maesobotrya cordulata | 328 | 1.5 | 13.3 |
| MAESPF | Phyllanthaceae | Maesobotrya floribunda | 28 | 0.3 | 2.2 |
| MAESBA | Phyllanthaceae | Maesobotrya griffoniana | 1,096 | 6.2 | 70.8 |
| MAESPU | Phyllanthaceae | Maesobotrya klaineana | 355 | 1.2 | 10.3 |
| MAESGF | Phyllanthaceae | Maesobotrya lonpipes | 13 | 0.1 | 0.3 |
| MAESGL | Phyllanthaceae | Maesobotrya pauciflora | 2 | 0.1 | 0.1 |
| PHYLDI | Phyllanthaceae | Phyllanthus diandrus | 448 | 2 | 19.2 |
| PROTMA | Phyllanthaceae | Protomegabaria macrophylla | 3 | 0.1 | 0.1 |
| PROT02 | Phyllanthaceae | Protomegabaria stapfiana | 3,001 | 54.4 | 607.2 |
| THECCB | Phyllanthaceae | Thecacoris leptobotrya | 96 | 0.2 | 0.8 |
| UAPA02 | Phyllanthaceae | Uapaca staudtii | 606 | 6.1 | 77 |
| CARPAL | Polygalaceae | Carpolobia gabonensis | 39 | 0.1 | 0.1 |
| DRYP02 | Putrangivaceae | Drypetes gabonensis | 1,428 | 0.6 | 1.6 |
| DRYP03 | Putrangivaceae | Drypetes ituriensis | 1 | 0.1 | 0.1 |
| LASIMA | Rhamnaceae | Lasiodiscus fasciculiflorus | 11 | 0.1 | 0.4 |
| MAESEM | Rhamnaceae | Maesopsis eminii | 1 | 0.1 | 0.1 |
| ANOPKL | Rhizophoraceae | Anopyxis klaineana | 5 | 0.1 | 0.1 |
| RUBI04 | Rhizophoraceae | Cassipourea dinklagei | 38 | 0.1 | 0.4 |
| AULAGL | Rubiaceae | Aulacocalyx subulata subsp. glabra | 4 | 0.1 | 0.1 |
| BELO02 | Rubiaceae | Belonophora coriacea | 2 | 0.1 | 0.3 |
| BERT02 | Rubiaceae | Bertiera sp. | 1 | 0.1 | 0.1 |
| BERT03 | Rubiaceae | Bertiera subsessilis | 2 | 0.1 | 0.1 |
| CHAZ02 | Rubiaceae | Chassalia pleuroneura | 14 | 0.1 | 0.1 |
| CHAZCM | Rubiaceae | Chazaliella cf. macrocarpa | 2 | 0.1 | 0.1 |
| COFF02 | Rubiaceae | Coffea mayombensis | 845 | 0.6 | 2.8 |
| CRATPF | Rubiaceae | Craterispermum caudatum | 63 | 0.1 | 0.3 |
| CRATSN | Rubiaceae | Craterispermum cerinanthum | 674 | 5 | 70.7 |
| CRATGF | Rubiaceae | Craterispermum gabonicum | 499 | 0.4 | 1.1 |
| CRATLE | Rubiaceae | Craterispermum ledermannii | 4 | 0.1 | 0.1 |
| CUVILO | Rubiaceae | Cuviera longiflora | 1 | 0.1 | 0.1 |
| CUVI02 | Rubiaceae | Cuviera physinodes | 126 | 0.1 | 0.1 |
| CUVIUN | Rubiaceae | Cuviera uncinula | 72 | 0.1 | 0.1 |
| EUCL02 | Rubiaceae | Euclinia longiflora | 77 | 0.1 | 0.1 |
| GAERSP | Rubiaceae | Gaertnera gabonensis | 306 | 0.1 | 0.2 |
| GAER02 | Rubiaceae | Gaertnera paniculata | 275 | 0.1 | 0.3 |
| HALLCI | Rubiaceae | Hallea ledermannii | 24 | 0.1 | 0.1 |
| HEINCR | Rubiaceae | Heinsia crinita | 20 | 0.1 | 0.1 |
| IXOR03 | Rubiaceae | Ixora aneimenodesma | 102 | 0.1 | 0.5 |
| IXOR02 | Rubiaceae | Ixora hippoperifera | 84 | 0.2 | 0.7 |
| IXORPF | Rubiaceae | Ixora praetermissa | 112 | 0.2 | 1 |
| LASYMA | Rubiaceae | Lasianthus mayumbensis | 1 | 0.1 | 0.1 |
| LASI02 | Rubiaceae | Lasianthus sp. | 1 | 0.1 | 0.1 |
| LEPTCA | Rubiaceae | Leptactina arnoldiana | 77 | 0.2 | 1 |
| MASSAC | Rubiaceae | Massularia acuminata | 521 | 2 | 17.2 |
| MASSST | Rubiaceae | Massularia stevartiana | 5 | 0.5 | 8.5 |
| NICHSO | Rubiaceae | Nichallea soyauxii | 27 | 0.2 | 1.5 |
| OXYAFO | Rubiaceae | Oxyanthus formosus | 53 | 0.6 | 4 |
| CUVIPC | Rubiaceae | Oxyanthus laxiflorus | 20 | 0.1 | 0.1 |
| PAURCA | Rubiaceae | Pauridiantha callicarpoides | 5 | 0.1 | 0.1 |
| PAUR02 | Rubiaceae | Pauridiantha mayumbensis. | 158 | 0.1 | 0.2 |
| PAURFC | Rubiaceae | Pauridiantha smetsiana | 90 | 0.1 | 0.1 |
| PAUSJO | Rubiaceae | Pausinystalia johimbe | 663 | 0.4 | 1.4 |
| PAUSMA | Rubiaceae | Pausinystalia macroceras | 115 | 0.4 | 1.6 |
| TARE02 | Rubiaceae | Pavetta corynbosa | 95 | 0.1 | 0.2 |
| PAVE02 | Rubiaceae | Pavetta macrophylla | 96 | 0.2 | 0.8 |
| PAVENB | Rubiaceae | Pavetta urophylla | 168 | 0.4 | 1.9 |
| PORTCL | Rubiaceae | Porterandia cladantha | 22 | 0.1 | 0.1 |
| POUCAF | Rubiaceae | Pouchetia africana | 1,319 | 0.6 | 2 |
| PSYC02 | Rubiaceae | Psychotria anthocleistifolia | 2 | 0.1 | 0.1 |
| PSYC05 | Rubiaceae | Psychotria dewildei | 1 | 0.1 | 0.1 |
| PSYCDE | Rubiaceae | Psychotria flagelliflora | 3 | 0.1 | 0.1 |
| PSYC04 | Rubiaceae | Psychotria sp.1 | 1 | 0.1 | 0.1 |
| PSYCFE | Rubiaceae | Psychotria sp.2 | 48 | 0.1 | 0.1 |
| RUBI03 | Rubiaceae | Psydrax splendens | 281 | 0.7 | 4.3 |
| ROTH02 | Rubiaceae | Rothmannia macrocarpa | 62 | 0.2 | 0.9 |
| RUBIPU | Rubiaceae | Schizocolea ochreata | 13 | 0.1 | 0.2 |
| SCHUMA | Rubiaceae | Schumanniophyton magnificum | 135 | 0.3 | 1.5 |
| TRYC02 | Rubiaceae | Sericanthe gabonensis | 481 | 1 | 4.8 |
| PAVEPF | Rubiaceae | Tarenna conferta | 15 | 0.1 | 0.2 |
| TAREJO | Rubiaceae | Tarenna jolinonii | 28 | 0.1 | 0.1 |
| TRYCFM | Rubiaceae | Tricalysia lasiodelphys | 146 | 1.1 | 7.9 |
| TRYC04 | Rubiaceae | Tricalysia micrantha | 161 | 1.1 | 6.9 |
| AULAJA | Rubiaceae | Tricalysia pangolina | 50 | 0.1 | 0.3 |
| TRYC03 | Rubiaceae | Tricalysia soyauxii | 912 | 3.8 | 25.2 |
| PSYC03 | Rubiaceae | Trichostachys longifolia | 1 | 0.1 | 0.1 |
| CASE02 | Salicaceae | Casearia barteri | 218 | 0.1 | 0.2 |
| HOMA03 | Salicaceae | Homalium africanum | 34 | 0.1 | 0.2 |
| HOMA02 | Salicaceae | Homalium le-testui | 141 | 0.2 | 1 |
| ONCOFL | Salicaceae | Oncoba flagelliflora | 618 | 6.3 | 80.7 |
| ONCOGL | Salicaceae | Oncoba glauca | 1 | 0.1 | 0.1 |
| TRICGA | Salicaceae | Trichostephanus gabonensis | 37 | 0.1 | 0.1 |
| OKOUAU | Santalaceae | Okoubaka aubrevillei | 1 | 0.1 | 0.1 |
| ALLO02 | Sapindaceae | Allophylus cobbe | 28 | 0.1 | 0.1 |
| BLIGWE | Sapindaceae | Blighia sapida | 2 | 0.1 | 0.1 |
| CHYTBC | Sapindaceae | Chytranthus gilletii | 7 | 0.1 | 0.1 |
| CHYT02 | Sapindaceae | Chytranthus macrobotrys | 76 | 0.1 | 0.1 |
| CHYTTA | Sapindaceae | Chytranthus talbotii | 45 | 0.1 | 0.1 |
| ERIOPU | Sapindaceae | Eriocoelum oblongum | 96 | 0.1 | 0.1 |
| ERIO02 | Sapindaceae | Eriocoelum petiolare | 842 | 0.5 | 3.4 |
| PANC03 | Sapindaceae | Haplocoelum gabonicum | 138 | 0.1 | 0.5 |
| LACC03 | Sapindaceae | Laccodiscus klaineanus | 21 | 0.1 | 0.1 |
| LACC02 | Sapindaceae | Laccodiscus pseudostipularis | 383 | 0.6 | 3.5 |
| CHYT03 | Sapindaceae | Lecaniodiscus cupanioides | 69 | 0.1 | 0.1 |
| CHYTGF | Sapindaceae | Lychnodiscus cf. brevibracteatus | 29 | 0.1 | 0.1 |
| PANC02 | Sapindaceae | Pancovia sp. nov. | 4,398 | 52.5 | 1195.6 |
| SAPI02 | Sapindaceae | Placodiscus caudatus | 724 | 1.7 | 10 |
| CHYTBA | Sapindaceae | Placodiscus opacus | 211 | 0.1 | 0.3 |
| BAILTO | Sapotaceae | Baillonella toxisperma | 1 | 0.1 | 0.1 |
| CHRYWE | Sapotaceae | Chrysophyllum welwitschii | 1 | 0.1 | 0.1 |
| ENGLST | Sapotaceae | Englerophytum stelechanthum | 361 | 0.2 | 0.5 |
| LETEDU | Sapotaceae | Gluema sp.nov | 1 | 0.1 | 0.1 |
| LECOBI | Sapotaceae | Lecomtedoxa biraudii | 31 | 0.2 | 1 |
| LECO02 | Sapotaceae | Lecomtedoxa sp. | 5 | 0.1 | 0.1 |
| OMPHEL | Sapotaceae | Omphalocarpum elatum | 2 | 0.2 | 2.6 |
| SYNC02 | Sapotaceae | Synsepalum cf. stipulatum | 266 | 0.2 | 0.3 |
| SYNC04 | Sapotaceae | Synsepalum dulcificum | 9 | 0.1 | 0.1 |
| SYNCPF | Sapotaceae | Synsepalum fleuryanum | 128 | 0.1 | 0.2 |
| SYNC03 | Sapotaceae | Synsepalum longecuneatum | 18 | 0.1 | 0.1 |
| SYNSSP | Sapotaceae | Synsepalum sp. | 5 | 0.1 | 0.1 |
| ZEREMA | Sapotaceae | Zeyherella mayumbensis | 686 | 4.9 | 40.1 |
| HANNKL | Simaroubaceae | Hannoa klaineana | 1 | 0.1 | 0.1 |
| ODYEGA | Simaroubaceae | Odyendyea gabonensis | 244 | 3.4 | 51 |
| QUASAF | Simaroubaceae | Quassia africana | 357 | 0.1 | 0.1 |
| LASIAF | Stemonuraceae | Lasianthera africana | 70 | 0.2 | 1.3 |
| UNKNOW | Unknown | Unknown Unknown | 2,109 | 7.1 | 60.3 |
| RINO03 | Violaceae | Rinorea gabunensis | 414 | 0.9 | 5.1 |
| RINO02 | Violaceae | Rinorea kamerunensis | 799 | 1.8 | 10.2 |
| RINOBC | Violaceae | Rinorea longisepala | 51 | 0.2 | 0.6 |
| ARGO02 | Violaceae | Rinorea sp. | 58 | 0.2 | 0.7 |
| ERISRE | Vochysiaceae | Erismadelphus exsul | 580 | 0.2 | 0.4 |
| ERIS02 | Vochysiaceae | Erismadelphus sp. | 78 | 0.1 | 0.1 |
| BALAWI | Zygophyllaceae | Balanites wilsoniana | 9 | 0.1 | 0.1 |
